# Internal Consistency and Floor/Ceiling Effects of the Gross Motor Function Measure for Use with Children Affected by Cancer: A Cross-Sectional Study

**DOI:** 10.3390/curroncol31090390

**Published:** 2024-09-06

**Authors:** Francesca Rossi, Monica Valle, Giovanni Galeoto, Marco Tofani, Paola Berchialla, Veronica Sciannameo, Daniele Bertin, Annalisa Calcagno, Roberto Casalaz, Margherita Cerboneschi, Marta Cervo, Annalisa Cornelli, Chiara Di Pede, Maria Esposito, Miriana Ferrarese, Paola Imazio, Maria Lorenzon, Lucia Longo, Andrea Martinuzzi, Gabriella Naretto, Nicoletta Orsini, Daniele Panzeri, Chiara Pellegrini, Michela Peranzoni, Fabiola Picone, Marco Rabusin, Federica Ricci, Claudia Zigrino, Giulia Zucchetti, Franca Fagioli

**Affiliations:** 1 Rehabilitation Service, Public Health and Paediatric Sciences Department, A.O.U. Città della Salute e della Scienza—Regina Margherita Children Hospital, 10126 Turin, Italy; 2 Department of Public Health and Pediatrics Sciences, University of Turin, 10124 Turin, Italy; monica.valle@unito.it (M.V.); m.espo79@libero.it (M.E.); federica.ricci@unito.it (F.R.); 3 Department of Human Neurosciences, Sapienza University of Rome, 00185 Rome, Italy; giovanni.galeoto@uniroma1.it; 4 Department of Life Sciences, Health and Allied Healthcare Professions, Università degli Studi “Link Campus University”, Via del Casale di San Pio V 44, 00165 Rome, Italy; m.tofani@unilink.it; 5 Management and Diagnostic Innovations & Clinical Pathways Research Area, Professional Development, Continuous Education and Research Service, IRCCS, Bambino Gesù Children’s Hospital, 00165 Rome, Italy; 6 Epidemiology and Public Health, Department of Clinical and Biological Sciences, University of Turin, 10124 Turin, Italy; paola.berchialla@unito.it (P.B.); veronica.sciannameo@unito.it (V.S.); 7 Pediatric Oncohematology, Stem Cell Transplantation and Cell Therapy Division, A.O.U. Città della Salute e della Scienza—Regina Margherita Children’s Hospital, 10126 Turin, Italy; daniele.bertin@unito.it (D.B.); giulia.zucchetti1@gmail.com (G.Z.); franca.fagioli@unito.it (F.F.); 8 Physical Therapy and Rehabilitation Department, Children’s Hospital Giannina Gaslini, 16147 Genoa, Italy; annalisacalcagno@gaslini.org (A.C.); nicoletta_orsini@yahoo.it (N.O.); 9 Paediatric Oncohematology Unit, Institute for Maternal and Child Health—IRCCS Burlo Garofolo, 34137 Trieste, Italy; roby.casalaz@hotmail.it (R.C.); marco.rabusin@burlo.trieste.it (M.R.); 10 Rehabilitation Department, IRCCS, Meyer Children’s Hospital, 50139 Firenze, Italy; m.cerboneschi@meyer.it (M.C.); marta.cervo@meyer.it (M.C.); fabiola.picone@meyer.it (F.P.); 11 Pediatric Oncology Department, ASST Papa Giovanni XXIII, 24127 Bergamo, Italy; annalisa.cornelli@gmail.com; 12 Neuromotor Rehabilitation Unit, Scientific Institute, IRCCS E. Medea, 31015 Conegliano, Italy; chiaradipede@yahoo.it (C.D.P.); maria.lorenzon@lanostrafamiglia.it (M.L.); andrea.martinuzzi@lanostrafamiglia.it (A.M.); 13 Health Professions of Rehabilitation Sciences Master’s Degree, Clinical and Biological Sciences Department, University of Turin, 10124 Turin, Italy; ferrarese.miriana@gmail.com (M.F.); longolucia95@gmail.com (L.L.); 14 Rehabilitation Department of Pediatric Orthopedics Unit, A.O.U. Città della Salute e della Scienza—Regina Margherita Children’s Hospital, 10126 Turin, Italy; paola.imazio@unito.it (P.I.); gabriella.naretto@libero.it (G.N.); 15 Neuro-Oncological Rehabilitation Unit, Scientific Institute IRCCS E. Medea, 23842 Bosisio Parini, Italy; daniele.panzeri@lanostrafamiglia.it; 16 Palliative Care, Pain Therapy and Rehabilitation Unit, Fondazione IRCCS Istituto Nazionale dei Tumori, 20133 Milan, Italy; chiara.pellegrini@istitutotumori.mi.it; 17 Department of Physiotherapy, Hospital of Bolzano, 39100 Bolzano, Italy; michela.peranzoni@sabes.it; 18 Unit for Severe Disabilities in Developmental Age and Young Adults (Developmental Neurology and Neurorehabilitation), Scientific Institute IRCCS “Eugenio Medea”, 72100 Brindisi, Italy; claudia.zigrino@lanostrafamiglia.it

**Keywords:** physical therapy, rehabilitative evaluation, GMFM-88, pediatric oncology

## Abstract

Children/adolescents with cancer can develop adverse effects impacting gross motor function. There is a lack of gross motor function assessment tools that have been validated for this population. The aim of this multicenter cross-sectional study was to preliminary validate the 88-item Gross Motor Function Measure (GMFM-88) for use in children/adolescents with cancer, exploring internal consistency and floor/ceiling effect. Inclusion criteria regarded children/adolescents diagnosed with cancer on treatment or <1 year off therapy. The internal consistency was assessed using Cronbach’s α, and the floor–ceiling effects were calculated through percentage. This study involved 217 participants with heterogeneous neoplasm conditions. Internal consistency was good, with a Cronbach’s α of 0.989. Floor–ceiling effect analysis reveals that several items obtained a dichotomous scoring distribution in each of the five sub-scales of the GMFM-88. This can be explained by the heterogeneous clinical characteristics of the target population. The preliminary validation of GMFM-88 in a group of children/adolescents affected by cancer suggests that some items are not able to discriminate between different gross motor function levels, and therefore it does not represent an informative tool to measure gross motor function in children with cancer. Future research is needed to define which ones could be more useful for clinical practice.

## 1. Introduction

Worldwide, the incidence of neoplasms corresponds to 16.5 cases each year for every 100,000 children, with more than half of these now surviving into adulthood [1]. Despite this positive prognosis, two-thirds of children diagnosed with cancer will develop at least one late or long-term adverse effect [2,3], related both to cancer itself and to cancer treatment. Physical functioning can be significantly impaired by various factors, especially in the context of malignant neoplasms. In children and adolescents with central nervous system (CNS) tumors, neurological deficits such as ataxia, apraxia, hemiparesis, sensory loss, spasticity, and cognitive impairments may occur [4]. Those with bone tumors often undergo surgeries like limb-salvage procedures, rotationplasty, or amputation, which can lead to complications such as wound dehiscence, pain, and restrictions on weight-bearing and activity [5]. Children with leukemia tend to have poorer gross and fine motor performance compared to their healthy peers [6]. Motor impairment can be exacerbated by antineoplastic treatments such as allogenic stem cell transplantation, which may result in lung, joint, and fascia impairments due to graft-versus-host disease [7]. Corticosteroid treatments, while necessary for managing certain aspects of cancer, can lead to reduced bone mineral density, osteoporosis, and osteonecrosis [8,9]. Some chemotherapy agents, such as vincristine, can induce peripheral neuropathy in children and adolescents, further hindering their physical functioning [10]. Similarly, anthracyclines, a class of chemotherapy drugs, are known for their cardiotoxic effects [11]. Moreover, CNS-penetrating chemotherapy or cranial radiation can result in long-term neurocognitive effects [12,13], and craniospinal irradiation may cause spinal deformities [14]. Hospitalization further compounds these challenges. Children with cancer tend to be less active during hospital stays than when they are at home [15], leading to increased bed rest. This inactivity can have severe consequences, including cardiovascular sequelae [16,17], decreased muscle mass, and reduced muscle strength [17,18]. These factors collectively contribute to a significant decline in the physical functioning of children and adolescents undergoing cancer treatment.

Rehabilitation is crucial for children and adolescents with cancer, as it plays a vital role in mitigating the physical and cognitive impairments caused by the disease and its treatment. Early and comprehensive rehabilitation can help improve mobility, enhance motor skills, and restore functional independence, allowing these young patients to maintain a higher quality of life during and after treatment. Although some studies conducted on the pediatric cancer population have recognized the importance of the role of rehabilitation in reducing motor complications and in promoting a better quality of life, more evidence is needed regarding the types of exercises that can be proposed [19,20]. To improve the rehabilitation care of pediatric cancer children/adolescents, it is therefore important to broaden the research by structuring multi-center trials, which allow the collection of longitudinal data [21] involving a larger population.

To ensure the effectiveness of rehabilitation programs, the use of adequate and standardized outcome measures is essential. These measures enable healthcare professionals to accurately assess progress, tailor interventions to individual needs, and ultimately optimize the long-term recovery and well-being of the pediatric cancer population. Therefore, objective, reliable, specific, and sensitive rehabilitation assessment tools are needed in order to define the functional status of the child/adolescent and to measure motor outcomes [21,22]. Since the psychometric properties of a measurement tool are closely linked to the specific population in which the measurement is used [23], it is necessary to adopt assessment tools that are validated for the childhood cancer population. Currently, some assessment tools have been already created for children/adolescents with cancer, to be used in the fields of physical exercise and rehabilitation [24] such as the Gross Motor Function Measure-Acute Lymphoblastic Leukemia (GMFM-ALL) [25], which investigates gross motor function; the Motor Performance in Pediatric Oncology (MOON) [26], which evaluates motor performance; the Pediatric modified Total Neuropathy Scale (Ped-mTNS) [27], which assesses chemotherapy-induced peripheral neuropathy’s signs and symptoms; and the Toronto Extremity Salvage Score (TESS) [28], which allows a subjective evaluation of gross motor function and disability in children/adolescents affected by musculoskeletal tumors. A recent scoping review regarding physical therapy interventions in childhood cancer [29] underlined how the most common outcome assessed in the selected studies was gross motor function. This is understandable considering the extent to which limited gross motor function can affect the child/adolescent’s ability to participate in age-appropriate activities, including play, with a consequently high relevance of this outcome for children/adolescents. Referring to assessment tools that investigate gross motor function in pediatric cancer patients, the TESS usability is limited to children/adolescents affected by musculoskeletal tumors. The GMFM-ALL is a reduced version of the Gross Motor Function Measure (GMFM-88) [29,30], a tool that has been used to evaluate gross motor function in various pediatric populations [31,32,33,34,35,36,37], including that of children/adolescents with cancer [38,39]. Anyway, the GMFM-ALL evaluates a high functional level (i.e., jump on one leg, jump off a step), which is not possessed by younger children, by those with major physical impairments, or during specific phases of cancer treatment, such as palliative care.

The need for a gross motor function assessment tool that allows evaluation of the broadest pediatric oncological population possible is a priority in order to appropriately measure a child/adolescent’s meaningful outcome, to homogenize future research studies, and to facilitate their comparison through meta-analysis processes. To enable their use in multicenter studies, it is preferable to choose assessment tools that use materials and equipment that are easy to find and of reasonable cost. The GMFM-88 is an assessment tool broadly known and used by many therapists who take care of pediatric children/adolescents, and it uses materials available in a common rehabilitation service. Indeed, due to the quantitative nature of the GMFM-88 in measuring gross motor function, this tool can be used with subjects whose gross motor function is compromised in different ways (e.g., by weakness, neuropathy, ataxia, hemiparesis, amputation), making it suitable for evaluating a broader population of children and adolescents affected by cancer. 

Therefore, due to the lack of gross motor function assessment tools validated for children and adolescents (age range: 6 months–18 years) affected by different kinds of cancer and undergoing various treatment phases, the goal of this multicenter study was to explore for the first time the internal consistency and floor/ceiling effects of the GMFM-88 when used for this population. Concerning the objective, the research group hypothesized that: (1) all the items of the GMFM-88 would positively contribute to determining the total score; and (2) some items of the GMFM-88 may not be clinically sensitive for the target population and can show floor–ceiling effect.

## 2. Materials and Methods

This article was prepared according to the Strengthening the reporting of observational studies in epidemiology (STROBE) checklist for cross-sectional studies [40].

### 2.1. Study Group

This observational multicenter study has been conducted by a research group composed of neuro and pscychomotricity therapists of developmental age (TNPEEs), physiotherapists, pediatric neuropsychiatrists, physiatrists, oncologists, and biostatisticians from the Rehabilitation Working Group of the Italian Association of Pediatric Hematology and Oncology (AIEOP) [41], in collaboration with the Rehabilitation and Outcome Measures Assessment (ROMA) Association. Thus, the research group has set up a multicenter cross-sectional study involving ten different hospitals, residential rehabilitation centers, outpatients’ services, and universities spread across all of Italy. This study was approved by the Ethical Committee of the coordinator center of this study (Comitato Etico Interaziendale AOU Città della Salute e della Scienza di Torino, AO Ordine Mauriziano e ASL Città di Torino) and by all the other Ethical Committees of the centers involved in this study. This study was a sub-study of a larger study designed to evaluate gross motor function in subjects of developmental age affected by cancer. This study was conducted according to the Declaration of Helsinki [42] and has been registered on Clinicaltrials.gov (NCT04862130).

### 2.2. Measure

The Gross Motor Function Measure, an 88-item measure also known as the GMFM-88, is a criterion-referenced observational measure specifically developed to evaluate changes in gross motor function over time in children with cerebral palsy [30,31]. The GMFM-88 has 5 dimensions: A—lying and rolling (17 items); B—sitting (20 items); C—kneeling and crawling (14 items); D—standing (13 items); and E—walking, running, and jumping (24 items). The items are scored from 0 to 3 [30] by the physical therapist/neuro and psychomotricity therapists of developmental age through direct observation of the child’s/adolescent’s motor performance. This measure is based on a reflective model that assumes that all items in a scale or subscale are manifestations of one underlying construct and are expected to be correlated [43]. The original GMFM-88 developers have been contacted and agreed to the investigation of internal consistency and floor–ceiling effect of the scale for a pediatric population affected by cancer.

### 2.3. Sampling 

Sample size was determined using two strategies: (a) analyzing previous validation studies and (b) defining the number according to the statistical analysis to be performed. Regarding the first strategy, sample size for GMFM-88 in populations other than cerebral palsy ranged from 73 for the brain injury population to 123 for the Down’s syndrome population, while in the original validation study [30], the sample involved 111 children with cerebral palsy. Regarding the power analyses, a minimum number of 50 participants is required to calculate internal consistency, thus avoiding the risk of bias due to insufficient populations [44]. However, considering that in Italy the cumulative risk of cancer in children is about 400 per million every year [1], and setting a confidence level of 95% and 5% margin of error, the minimum sample size required is 197 participants. Using a twin-track approach for sampling guaranteed relevance for both clinical and research purposes, the research group has therefore decided to use 197 as the minimum sample size.

### 2.4. Data Collection and Procedures

The study involved ten Italian centers, some belonging to the AIEOP network, and other external centers that take care of these children/adolescents in the community. Before starting the study, a training package for using the GMFM-88 was organized for the AIEOP Rehabilitation working group members. Six hours of frontal training were performed by a qualified physical therapist. Subsequently, to foster more confidence with GMFM-88 scoring and administration, healthcare professionals were involved in a supervised practical examination session in which they scored some videotaped administrations of the GMFM-88. Furthermore, a specific training session on methodological issues was also provided. This promoted a homogeneous level of confidence with the GMFM-88 and procedures.

Individuals that satisfied the inclusion criteria were recruited from inpatient and outpatient services of Regina Margherita Children’s Hospital—Torino, Giannina Gaslini Children’s Hospital—Genova, Meyer Children’s Hospital—Firenze, Papa Giovanni XXIII Hospital—Bergamo, Hospital of Bolzano, IRCCS E. Medea of Bosisio Parini (LC), Brindisi and Conegliano (TV), IRCCS Istituto Nazionale dei Tumori—Milano, and IRCCS Burlo Garofolo—Trieste. The recruitment period lasted from November 2019 to November 2021. Data regarding subjects’ socio-demographic and oncological characteristics were collected through a paper case report form, then inserted in an Excel dataset and subsequently analyzed from December 2021 to November 2022. The convenience sample met the following inclusion criteria: (1) to be children or adolescents (age range 6 months–18 years), and (2) to have a confirmed diagnosis of cancer. Exclusion criteria regarded not to be able to collaborate in simple requests.

Recruitment strategies included a retrospective chart review to identify rehabilitative evaluations carried previously with the GMFM-88 in every participant center and to verify the fulfillment of the inclusion criteria, and the recruitment of all new children/adolescents with cancer referred to rehabilitation services. The review of the past GMFM-88 scores and the data entry were performed by the data manager of each center. The prospective sample was evaluated at the referral to the rehabilitation service. The study presentation was made by phone call or through face-to-face meetings with children and families within the participating centers, and with the use of brochures to explain the study purposes and data management strategies. Informed written consent was signed by parents/guardians and children/adolescents who agreed to participate. 

### 2.5. Data Analysis

Socio-demographic information was analyzed with descriptive statistics, using frequency, mean (SD), and median (IQR) (when appropriate). With regards to the first hypothesis, Cronbach’s α was used to evaluate the internal consistency of each subscale of the GMFM-88. Our hypothesis anticipated a correlation between items of each subscale. As reported by Nunnally [44], a satisfactory index of a scale’s homogeneity should have an α coefficient ≥0.70. We also measured correlation between each GMFM-88 dimension using the Pearson correlation coefficient. The Pearson correlation coefficient ranges from 0 (indicating no linear relationship) to 1 (indicating a perfect linear relationship) and was interpreted as follows: <0.3 indicates a weak relationship; 0.3–0.69 indicates a moderate relationship; and ≥0.7 indicates a strong relationship. The correlation values can be either positive or negative, indicating the direction of the relationship [45].

With regards to the second hypothesis, the floor–ceiling effect was calculated for each item of the GMFM-88. The floor–ceiling effect describes whether participants have scores that are at, or near, the possible lower or upper limits, respectively, preventing measurement of variance above or below a certain level. Floor and ceiling effects were evaluated by determining the proportion of patients who achieved the highest and lowest scores, and effects were considered present if 15% of patients obtained either the lowest or highest possible score [45]. Floor and ceiling effects have been classified as significant if ≥15%, moderate if 10% to <15%, minor if 5% to <10%, and negligible if <5% of participants score the lowest or highest possible score on a measure [46].

## 3. Results

The GMFM-88 was administered to 217 children (46.5% F–53.5% M) with a median age of 6.9 years (IQR 2.7–11.3). Males composed 53.3% of the sample; the diagnosis of CNS tumors was reported in 55.8% of the subjects, and the more represented treatment phase was to be on treatment (66.8%). Sample characteristics are summarized in Table 1. 

Internal consistency estimates revealed a Cronbach’s α of 0.989 for the total score of the GMFM-88, while for each dimension, the Cronbach’s α was of 0.953 for Dimension A, of 0.957 for Dimension B, of 0.965 for Dimension C, of 0.966 for Dimension D, and of 0.974 of for Dimension E. Item-total statistics for each subscale of the GMFM-88 and each dimension are reported in Table 2.

The analysis of the correlation of the GMFM-88 dimension, as measured with the Pearson correlation coefficient, revealed a strong positive linear and significant correlation with GMFM-88 with each subscale (ranging from 0.839 to 0.937) and in between each dimension (ranging from 0.671 to 0.917). Results are summarized in Table 3.

The floor–ceiling effect was calculated for both GMFM-88 subscales and for each item of the GMFM-88. In particular, dimensions A and B revealed the ceiling effect, dimension C displayed both the ceiling and floor effect, while dimensions D and E showed the floor effect. Results for GMFM-88 dimensions are synthesized in Table 4, while results for each item are summarized in Supplementary Tables S1–S4.

**Figure 1:**
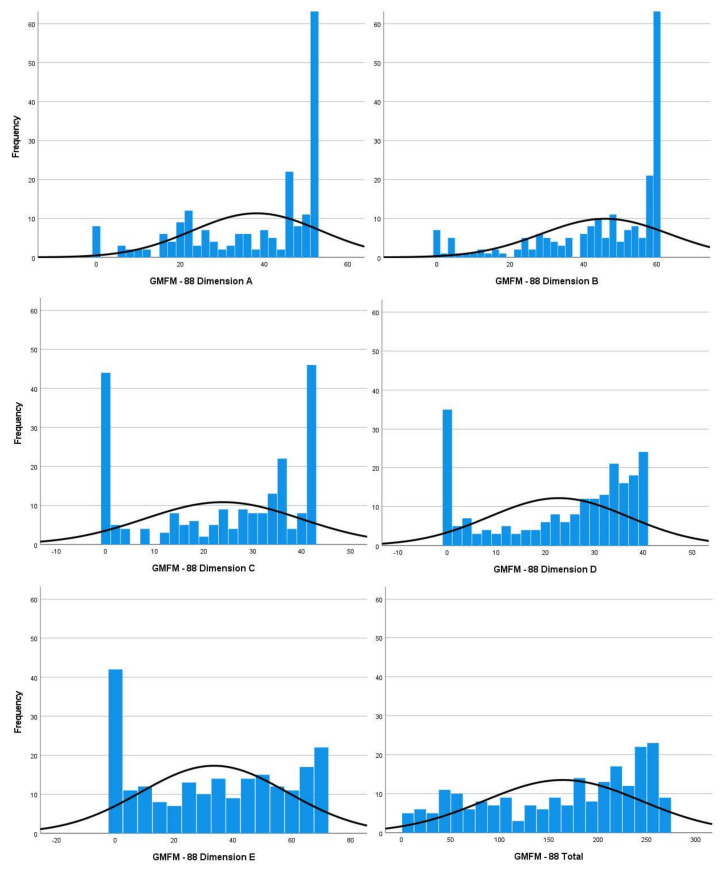
Score distribution of each GMFM-88 dimension and total score.

To better understand the score distribution of each GMFM-88 dimension and the total score, results are also represented in Figure 1, with evidence of skewness and kurtosis.

## 4. Discussion

The aim of this study was to explore the internal consistency and floor–ceiling effect of GMFM-88 in a pediatric oncological population in light of the relevant role of gross motor function for independence and quality of life and the lack of validated assessment tools to quantify this aspect.

Internal consistency of the GMFM-88 in a group of 217 Italian children and younger adolescents with cancer showed a high coefficient of Cronbach’s α, both for the whole test and for each dimension of the scale. It was found a strong positive linear and significant correlation with GMFM-88 with each subscale and in between each dimension. The analysis of the scoring distribution reveals floor and ceiling effects in several items and in each of the GMFM five dimensions.

For what concerns internal consistency, we found high values of Cronbach’s alpha ranging from 0.953 to 0.989. This finding is in line with validation of the modified version of the GMFM-88 for children with both spastic diplegia and visual impairment, reporting internal consistency of dimension scores between 0.97 and 1.00 [47], or with the Korean version [48,49] of the scale but slightly higher than the Indonesian version (alpha range 0.79–0.89 [50]). Not in all the validation studies is reported in the internal consistency analysis. However, a high coefficient α does not always mean a high degree of internal consistency [51]; in fact, this result might suggest that some items may be redundant since they are probably testing the same aspect but simply in a different way. Some authors report that acceptable values of α should range from 0.70 to 0.95 [52], even though a maximum α value of 0.90 is recommended [23]. Since the coefficient α was higher than 0.90 for all dimensions in our study, this could be due to length of scale, and it is possible that the number of items would need to be reduced to achieve better internal consistency for the target population. 

One important component of this validation was the identification of floor and ceiling effects, which indicate the ability of the GMFM88 to distinguish between respondents at the extreme ends of the scale [53]. Floor and ceiling effects are defined as the proportion of respondents scoring the highest (ceiling) or lowest (floor) possible score across any given domain, measuring the sensitivity and coverage of a questionnaire at each end of the scale. For example, if a large proportion of patients receive the lowest possible score on a questionnaire, then that suggests that all of those patients have the same level of health, which in turn indicates the inability of that instrument to differentiate among those at the low end of the spectrum [54]. The high floor–ceiling effect also may suggest limited instrument range, measurement inaccuracy, and response bias [55], all of which indicate the inaccuracy of an assessment tool. 

Although a floor or ceiling effect was not observed for the total GMFM-88 score, our findings emphasize that when the GMFM-88 dimensions are analyzed separately, three out of five dimensions exhibit floor and ceiling effects. In particular, the two dimensions that revealed the floor effect are those that investigate a low functional level related to abilities in “Lying and Rolling” (dimension A) and “Sitting” (dimension B). The abilities investigated in these dimensions are often not allowed during the post-surgical phase or compromised in palliative care settings or in children affected by neurological impairments, being therefore impaired only in a small portion of our sample. “Crawling and kneeling” (dimension C) group a medium functional level of abilities that can present different levels of issues both for younger children and for those affected by muscular weakness, neurological deficits, or in subjects with bone tumors who undergo surgeries. It is therefore reasonable that in our population it reached both floor and ceiling effects. “Standing” (dimension D) and “Walking, running and jumping” (dimension E) dimensions contain high-level abilities that can be compromised in many subjects during the active phase of treatment, justifying these results in our sample.

Furthermore, the analysis of the scoring distribution within each item showed that out of eighty-eight items, a floor–ceiling effect was revealed for all scoring categories in eight items, for three of four scoring categories in ten items, for two of them in forty-four items, and for one scoring category in seven items, while only one item did not show a floor–ceiling effect (please see Supplementary Materials). 

Specifically, eight items—three in dimension A “lying and rolling” and five in dimension B “sitting”—demonstrated floor–ceiling effects across all scoring categories, where scores of 0 to 2 showed a floor effect and a score of 3 indicated a ceiling effect. These results suggest that the detected abilities are not so frequently impaired in the target population, thus being poorly informative on the functional level of the child/adolescent. Referring to dimension A (Supplementary Table S1), these items were: item 1 “supine, head in midline: turns head with extremities symmetrical,” and items 6 and 7 “supine, reaches out with right/left arm, hand crosses midline toward toy”. The ability required in item 1 can be impaired in children/adolescents with CNS lesions and spasticity with the activation of abnormal synergies, leading to difficulties in dissociating movements [56]. Most of the children/adolescents with cancer and CNS involvement are those with CNS tumors, and the most common malignant brain tumor of childhood is medulloblastoma, accounting for 20% of all primary CNS tumors in children/adolescents younger than 19 years old [57]. The main impairments in these children/adolescents are due to hypotonia and ataxia, with no spasticity or activation of pathological synergies. The ability to cross midline with arms (items 6 and 7) can be primarily impaired in children with CNS cancers affected by spasticity or by very significant muscle weakness, in children/adolescents affected by shoulder osteonecrosis, or in children/adolescents with upper limb bone tumors. Rates of symptomatic osteonecrosis in children affected by leukemia range from <1% to 18%, with a greater incidence in adolescence [58] and in the femoral head [59], but the literature is scarce on the prevalence of osteonecrosis of the shoulders in these children/adolescents [60]. Osteosarcomas make up 40% of bone tumors and 2% of neoplastic cases in children, with an incidence rate of 0.33 cases/year/100,000 children [1], and they most commonly happen in the long bones around the knee. Due to the rarity of the cited conditions, it is understandable that only a small part of our sample was unable to perform these activities.

In dimension B (Supplementary Table S2), the starting position of the five items that showed a floor–ceiling effect for all scoring categories is sitting on the mat. Items 21 and 22 require children/adolescents to lift the head upright and maintain the position for 3 and 10 s, respectively, with chest support from the therapist. Items 23 and 24 require children/adolescents to sit on the mat with arm(s) propping for 5 s and arms free for 3 s. From a motor development point of view, the abilities to lift head in the described position and to maintain the sitting position independently are acquired very early in children’s development, the first one very much earlier than the age established as an inclusion criterion for the study population, and the second one between 6 and 9 months [61]. The prevalence of cancer in children under one year of age is less than 200 per million every year [1], therefore this age group was a minority in our sample. Two of the most common reasons a participant might not be able to perform these items are a medical recommendation not to maintain the sitting position in the post-surgical phase or being in the functional recovery phase after surgical removal of a CNS tumor. The difficulty in performing items 21 to 24 is therefore limited to a specific age range, treatment phase, and oncological diagnosis. In item 26, the child seated on the mat is required to touch a toy placed at 45° behind his/her side; this activity, which is feasible by children that have acquired a dynamic control of the sitting position, can be impaired in our population by the conditions mentioned above and in children/adolescents with shoulder osteonecrosis, upper limb bone tumors, or in those with CNS tumors and hemiparesis. These characteristics can be found in a small number of participants within our sample, as underlined by the ceiling and floor effect displayed in different scoring categories.

Other items, distributed in the five dimensions, showed a floor effect for scoring 1 and 2. Referring to Dimension A, an interesting finding was that all the items that require achieving the prone position (items 8 and 9), or which must be performed in prone position, gained a dichotomous score of 0 or 3 points. This result could be related to some aspects closely related to our population’s features: the prone position cannot be maintained in the post-surgical period by children/adolescents affected by CNS cancers with ventriculo-peritoneal derivation, and a lot of children/adolescents affected by cancer prefer not to stay in this position during treatment due to the pressure on the central venous catheter. 

One item in Dimension C “crawling and kneeling” (Supplementary Table S3) and six items of Dimension D “standing” (Supplementary Table S4) achieved a floor ceiling effect only for one score category, while one item in dimension E “walking, running and jumping” did not show a floor ceiling effect. Item 40 of Dimension C requires reaching the sitting position with free arms, starting from a quadrupedal position. Items of Dimension D involve two positions lifting left/right foot in standing position, arms free for 10 s (items 57/58), and three position changes such as attaining a standing position from a chair without using arms (item 59), attaining a standing position from right/left half kneeling (item 60/61) without using arms, and lowering to sit on the floor, arms free, starting from a standing position (item 62). Item 80 of Dimension E (Supplementary Table S5) regards the ability to jump. All these activities require strength, balance, and adequate range of movement in lower limb joints, which result in challenging and therefore more discriminant of different functional levels in children and adolescents affected by cancer. 

### Strengths and Limitations

The value of this study resides in its evaluation of internal consistency and floor/ceiling effect of the GMFM-88 in a population lacking validated gross motor function assessment tools. However, the research also has some limitations. The sample was composed of a reduced number of infants and children/adolescents affected by bone tumors, with a prevalence of participants with CNS cancers and undergoing active treatment. The external validity of the study can be seen as the suggestion of a more aware use of this assessment tool in the pediatric oncological population, considering that some items have been shown as redundant. Finally, the GMFM-88 requires a very long time to administer, due to the high number of items; our results suggest that some of the items are poorly informative and could be excluded. For example, items 46/47 “crawls up 4 steps on hands and knees/feet and crawls backwards down 4 steps on hands and knees/feet” are too easy for subjects with a medium level of weakness or for those affected by chemotherapy induced neuropathy, not feasible for subjects in the post-surgical phase or amputees, and of little meaning for school-age children and adolescents. Furthermore, a possible explanation of different scoring in items was provided by considering the heterogeneity of the sample, so that it would be useful to analyze how different items work across specific diagnostic groups. Only a few psychometric properties were measured, while, for instance, other parameters for reliability as well as validity (i.e., Rasch analysis) could help to better understand if the GMFM-88 is informative for pediatric population with cancer. At the end, in our study, a factor analysis was not performed, while it would be useful investigating the structural validity and multidimensionality of the scale. Factor analysis can also better explain how GMFM-88 works for children with neoplasm conditions. Future studies should consider these aspects.

## 5. Conclusions

The GMFM-88 is a widely used tool in different pediatric populations, both for research and clinical purposes, with the added value of using materials available in a common rehabilitation service. However, the preliminary validation of the GMFM-88 in a group of Italian children and adolescents affected by cancer suggests that some items may be redundant. Furthermore, scoring of several items showed a dichotomous distribution. However, floor and ceiling effects can indicate insufficient content validity and can result in insufficient reliability [62]. Future research should be directed at exploring the content validity of each item of the scale for the pediatric oncology population to establish which items could better discriminate between different levels of gross motor function and to obtain better internal consistency for the target population. This aim could be gained using the Content Validity Ratio with the involvement of an expert panel in order to define how much each item is relevant for the given population. This process could assist in the definition of a final panel of items that can be judged as clinically important for this specific population and relevant to describe the subject change over time. Other attempts to reduce the number of items on the GMFM-88 have already been performed in the effort to improve the interpretability and clinical usefulness of the GMFM, such as the development of the GMFM-66 [63]. At the end, the GMFM-88 seems not to be the best assessment tool to measure gross-motor function in children with neoplasm conditions, especially for the ceiling and floor effect and for the timing required for administering the scale.

## Figures and Tables

**Table 1:** Demographic characteristics of the sample (total 217).

Age	Years (IQR *)
Age to evaluation	6.9 years [IQR 2.7–11.3]
Gender	N (%)
Female	101 (46.5)
Male	116 (53.5)
Type of cancer	N (%)
Central nervous system tumors	121 (55.8)
Bone cancers	12 (5.5)
Leukemia/lymphoma	63 [29]
Other solid tumors	21 (9.7)
Treatment phase	N (%)
On treatment	145 (66.8)
Off therapy	31 (14.3)
Post-surgery	41 (18.9)

* IQR = interquartile range.

**Table 2:** Mean (SD) score and item-total statistics of GMFM-88.

GMFM88 ITEM	Mean	SD	Scale Mean If Item Deleted	Scale Variance If Item Deleted	Corrected Item-Total Correlation	Cronbach’s Alpha If Item Deleted
DIMENSION A						
1	2.69	0.868	35.502	220.436	0.502	0.953
2	2.57	0.995	35.613	215.183	0.626	0.952
3	2.50	1.043	35.682	216.533	0.548	0.953
4	2.60	0.894	35.585	218.373	0.578	0.952
5	2.62	0.876	35.567	216.423	0.670	0.951
6	2.75	0.810	35.438	218.784	0.627	0.952
7	2.68	0.913	35.507	215.649	0.670	0.951
8	2.31	1.169	35.876	205.832	0.813	0.948
9	2.28	1.173	35.899	206.230	0.797	0.949
10	2.23	1.284	35.949	204.974	0.757	0.949
11	2.05	1.329	36.134	201.283	0.833	0.948
12	1.86	1.421	36.332	199.001	0.832	0.948
13	1.88	1.422	36.313	199.012	0.830	0.948
14	2.06	1.383	36.115	199.102	0.857	0.947
15	2.07	1.365	36.106	199.206	0.866	0.947
16	1.53	1.472	36.664	201.835	0.727	0.950
17	1.52	1.469	36.668	202.621	0.709	0.951
DIMENSION B						
18	2.57	1.018	43.370	283.481	0.613	0.956
19	2.17	1.301	43.773	272.204	0.737	0.955
20	2.21	1.280	43.727	273.706	0.713	0.955
21	2.82	0.695	43.120	290.469	0.615	0.957
22	2.75	0.773	43.190	289.187	0.599	0.957
23	2.78	0.776	43.162	286.964	0.684	0.956
24	2.70	0.887	43.236	282.935	0.731	0.955
25	2.57	0.962	43.370	279.834	0.770	0.955
26	2.62	0.971	43.319	278.711	0.798	0.954
27	2.58	1.008	43.361	277.395	0.807	0.954
28	2.01	1.338	43.926	270.822	0.747	0.955
29	2.07	1.312	43.870	271.230	0.754	0.955
30	1.94	1.397	43.995	270.358	0.723	0.955
31	2.05	1.375	43.889	269.588	0.754	0.955
32	2.12	1.345	43.819	270.037	0.762	0.955
33	1.94	1.328	43.995	271.577	0.735	0.955
34	2.52	1.043	43.421	278.496	0.745	0.955
35	2.14	1.329	43.801	270.597	0.758	0.955
36	1.75	1.440	44.185	269.631	0.715	0.955
37	1.62	1.439	44.324	269.904	0.710	0.956
DIMENSION C						
38	2.69	0.868	22.424	222.079	0.714	0.964
39	2.57	0.995	21.687	221.587	0.820	0.962
40	2.50	1.043	21.977	221.634	0.821	0.962
41	2.60	0.894	21.954	219.979	0.785	0.962
42	2.62	0.876	21.945	218.784	0.850	0.961
43	2.75	0.810	21.931	218.157	0.865	0.961
44	2.68	0.913	21.889	219.293	0.822	0.961
45	2.31	1.169	21.968	219.087	0.826	0.961
46	2.28	1.173	22.839	226.108	0.649	0.965
47	2.23	1.284	22.959	227.623	0.633	0.965
48	2.05	1.329	21.917	219.076	0.881	0.960
49	1.86	1.421	22.263	218.056	0.841	0.961
50	1.88	1.422	22.286	218.325	0.835	0.961
51	2.06	1.383	22.286	217.835	0.837	0.961
DIMENSION D						
52	2.69	0.868	20.631	172.438	0.798	0.964
53	2.57	0.995	20.392	175.702	0.799	0.964
54	2.50	1.043	20.756	170.389	0.854	0.962
55	2.60	0.894	20.770	170.799	0.840	0.963
56	2.62	0.876	20.581	171.393	0.876	0.962
57	2.75	0.810	21.502	176.566	0.723	0.966
58	2.68	0.913	21.488	176.288	0.745	0.965
59	2.31	1.169	20.857	171.549	0.851	0.963
60	2.28	1.173	21.313	174.161	0.799	0.964
61	2.23	1.284	21.313	174.661	0.793	0.964
62	2.05	1.329	21.221	173.034	0.829	0.963
63	1.86	1.421	21.207	172.008	0.807	0.964
64	1.88	1.422	20.926	169.439	0.866	0.962
DIMENSION E						
65	2.69	0.868	31.276	577.794	0.728	0.973
66	2.57	0.995	31.276	577.794	0.728	0.973
67	2.50	1.043	31.147	585.311	0.642	0.974
68	2.60	0.894	31.332	574.112	0.767	0.973
69	2.62	0.876	31.410	570.854	0.808	0.973
70	2.75	0.810	31.548	566.360	0.839	0.972
71	2.68	0.913	32.088	567.460	0.793	0.973
72	2.31	1.169	31.668	565.426	0.834	0.973
73	2.28	1.173	32.111	567.228	0.834	0.973
74	2.23	1.284	32.558	579.109	0.749	0.973
75	2.05	1.329	32.180	565.630	0.849	0.972
76	1.86	1.421	32.189	565.534	0.851	0.972
77	1.88	1.422	32.631	580.706	0.704	0.973
78	2.06	1.383	31.677	563.784	0.850	0.972
79	2.07	1.365	31.677	563.951	0.847	0.972
80	1.53	1.472	32.714	584.529	0.789	0.973
81	1.52	1.469	32.618	579.024	0.760	0.973
82	2.57	1.018	32.866	590.014	0.665	0.974
83	2.17	1.301	32.876	590.517	0.657	0.974
84	2.21	1.280	32.069	569.352	0.783	0.973
85	2.82	0.695	32.212	569.168	0.822	0.973
86	2.75	0.773	32.585	577.086	0.747	0.973
87	2.78	0.776	32.599	577.408	0.748	0.973
88	2.70	0.887	32.714	580.149	0.701	0.974

**Table 3:** Correlation of the GMFM-88 dimensions.

GMFM-88 DIMENSIONS						
	A	B	C	D	E	TOTAL
A	1	0.787 **	0.734 **	0.671 **	0.674 **	0.839 **
B		1	0.837 **	0.817 **	0.752 **	0.916 **
C			1	0.834 **	0.798 **	0.920 **
D				1	0.917 **	0.937 **
E					1	0.927 **
TOTAL						1

** *p* < 0.01.

**Table 4:** Floor–ceiling effect of GMFM-88 dimensions.

GMFM-88	Min–Max	Mean (SD)	Floor Effect N (%)	Ceiling Effect N (%)	Skewness	Kurtosis
Dimension A	0–51	38.18 (15.31)	8 (3.67)	81 (37.33) *	−0.97	−0.29
Dimension B	0–60	45.87 (17.47)	7 (3.23)	75 (34.56) *	−1.27	0.62
Dimension C	0–42	23.87 (15.97)	44 (20.28) *	41 (18.89) *	−0.42	−1.37
Dimension D	0–39	22.75 (14.23)	35 (16.13) *	24 (11.06)	−0.54	−1.25
Dimension E	0–72	33.48 (24.99)	38 (17.51) *	11 (5.07)	0.01	−1.41
Total	0–264	164.15 (80.06)	1 (0.46)	11 (5.07)	−0.47	−1.16

* Floor–ceiling effect.

## Data Availability

The data presented in this study are available on request from the corresponding author due to privacy reasons.

## Supplementary Materials

The following supporting information can be downloaded at: https://www.mdpi.com/article/10.3390/curroncol31090390/s1, Table S1: Ceiling–Floor Effect for Dimension B of the GMFM-88 (sample n = 217). Table S2: Ceiling–Floor Effect for Dimension C of the GMFM-88 (sample n = 217). Table S3: Ceiling–Floor Effect for Dimension D of the GMFM-88 (sample n = 217). Table S4: Ceiling–Floor Effect for Dimension E of the GMFM-88 (sample n = 217). Table S5: Ceiling-Floor Effect Dimension E of the GMFM-88.
