# Supplementary material for: Internal Consistency and Floor/Ceiling Effects of the Gross Motor Function Measure for Use with Children Affected by Cancer: A Cross-Sectional Study
Source: Curr Oncol. 2024 Sep 6;31(9):5291–306. doi: 10.3390/curroncol31090390 (PMC11430713; doi:10.3390/curroncol31090390)
Supplement: Supplementary file 1 [file curroncol-31-00390-s001.zip › curroncol-3105792-supplementary.pdf]

Current Oncology

Supplementary Materials

Table S1. Ceiling-floor effect Dimension A of the GMFM-88.

Ceiling-Floor Effect Dimension A of the GMFM-88 (sample 217)			
Dimension A	Response	Frequency	Percentage
Item 1	0	19	8.8*
	1	4	1.8*
	2	4	1.8*
	3	190	87.6**
Item 2	0	25	11.5*
	1	5	2.3*
	2	8	3.7*
	3	179	82.5
Item 3	0	27	12.4*
	1	9	4.1*
	2	9	4.1*
	3	172	79.3
Item 4	0	18	8.3*
	1	6	2.8*
	2	21	9.7*
	3	172	79.3
Item 5	0	17	7.8*
	1	6	2.8*
	2	20	9.2*
	3	174	80.2
Item 6	0	16	7.4*
	1	2	.9*
	2	3	1.4*
	3	196	90.3**
Item 7	0	21	9.7*
	1	3	1.4*
	2	1	.5*
	3	192	88.5**
Item 8	0	38	17.5
	1	10	4.6*
	2	16	7.4*
	3	153	70.5
Item 9	0	39	18.0
	1	9	4.1*
	2	20	9.2*
	3	149	68.7

Item 10	0	51	23.5
	1	5	2.3*
	2	3	1.4*
	3	158	72.8
Item 11	0	60	27.6
	1	5	2.3*
	2	16	7.4*
	3	136	62.7
Item 12	0	77	35.5
	1	6	2.8*
	2	6	2.8*
	3	128	59.0
Item 13	0	77	35.5
	1	4	1.8*
	2	6	2.8*
	3	130	59.9
Item 14	0	66	30.4
	1	1	.5*
	2	2	.9*
	3	148	68.2
Item 15	0	63	29.0
	1	4	1.8*
	2	3	1.4*
	3	147	67.7
Item 16	0	103	47.5
	1	2	.9*
	2	8	3.7*
	3	104	47.9
Item 17	0	103	47.5
	1	2	.9*
	2	9	4.1*
	3	103	47.5

* floor effect; ** ceiling effect

Table S2. Ceiling-Floor Effect Dimension B of the GMFM-88.

**Ceiling-Floor Effect Dimension B of the GMFM-88 (sample 217)**			
Dimension B	Response	Frequency	Percentage
Item 18	0	27	12.4*
	1	4	1.8*
	2	4	1.8*
	3	182	83.9
Item 19	0	55	25.4
	1	5	2.3*
	2	8	3.7*
	3	149	68.7
Item 20	0	52	24.4
	1	5	2.3*
	2	7	3.2*
	3	153	70.5
Item 21	0	12	5.5*
	1	3	1.4*
	2	0	0*
	3	202	93.1**
Item 22	0	13	6.0*
	1	5	2.3*
	2	5	2.3*
	3	194	89.4**
Item 23	0	15	6.9*
	1	1	.5*
	2	1	.5*
	3	200	92.2**
Item 24	0	20	9.2*
	1	2	.9*
	2	0	0*
	3	195	89.9**
Item 25	0	24	11.1*
	1	1	.5*
	2	19	8.8*
	3	173	79.7
Item 26	0	25	11.5*
	1	1	.5*
	2	5	2.3*
	3	186	85.7**
Item 27	0	27	12.4*
	1	2	.9*

	2	6	2.8*
	3	182	83.9
Item 28	0	63	29.0
	1	5	2.3*
	2	17	7.8*
	3	132	60.8
Item 29	0	59	27.2
	1	4	1.8*
	2	19	8.8*
	3	135	62.2
Item 30	0	72	33.2
	1	3	1.4*
	2	9	4.1*
	3	133	61.3
Item 31	0	65	30.0
	1	6	2.8*
	2	1	.5*
	3	145	66.8
Item 32	0	60	27.6
	1	6	2.8*
	2	1	.5*
	3	150	69.1
Item 33	0	65	30.0
	1	4	1.8*
	2	28	12.9*
	3	120	55.3
Item 34	0	29	13.4*
	1	3	1.4*
	2	11	5.1*
	3	174	80.2
Item 35	0	58	26.7
	1	3	1.4*
	2	6	2.8*
	3	150	69.1
Item 36	0	84	38.7
	1	7	3.2*
	2	6	2.8*
	3	120	55.3
Item 37	0	92	42.4
	1	7	3.2*
	2	12	5.5*

	3	106	48.8

* floor effect; ** ceiling effect

Table S3. Ceiling-Floor Effect Dimension C of the GMFM-88.

**Ceiling-Floor Effect Dimension C of the GMFM-88 (sample 217)**			
Dimension C	Response	Frequency	Percentage
Item 38	0	104	47.9
	1	9	4.1*
	2	7	3.2*
	3	97	44.7
Item 39	0	54	24.9
	1	5	2.3*
	2	5	2.3*
	3	153	70.5
Item 40	0	64	29.5
	1	3	1.4*
	2	42	19.4
	3	108	49.8
Item 41	0	75	34.6
	1	3	1.4*
	2	4	1.8*
	3	135	62.2
Item 42	0	69	31.8
	1	4	1.8*
	2	18	8.3*
	3	126	58.1
Item 43	0	69	31.8
	1	3	1.4*
	2	17	7.8*
	3	128	59.0
Item 44	0	69	31.8
	1	4	1.8*
	2	6	2.8*
	3	138	63.6
Item 45	0	72	33.2
	1	5	2.3*
	2	12	5.5*
	3	128	59.0
Item 46	0	135	62.2
	1	9	4.1*
	2	4	1.8*
	3	69	31.8
Item 47	0	144	66.4
	1	9	4.1*

	2	3	1.4*
	3	61	28.1
Item 48	0	61	28.1
	1	9	4.1*
	2	26	12.0*
	3	121	55.8
Item 49	0	90	41.5
	1	4	1.8*
	2	24	11.1*
	3	99	45.6
Item 50	0	91	41.9
	1	5	2.3*
	2	24	11.1*
	3	97	44.7
	0	92	42.4
Item 51	1	7	3.2*
	2	17	7.8*
	3	101	46.5

*floor effect

Table S4. Ceiling-Floor Effect Dimension D of the GMFM-88.

**Ceiling-Floor Effect Dimension D of the GMFM-88 (sample 217)**			
Dimension D	Response	Frequency	Percentage
Item 52	0	59	27.2
	1	7	3.2*
	2	1	.5*
	3	150	69.1
Item 53	0	41	18.9
	1	6	2.8*
	2	5	2.3*
	3	165	76.0
Item 54	0	63	29.0
	1	10	4.6*
	2	10	4.6*
	3	134	61.8
Item 55	0	64	29.5
	1	9	4.1*
	2	12	5.5*
	3	132	60.8
Item 56	0	53	24.4
	1	3	1.4*
	2	16	7.4*
	3	145	66.8
Item 57	0	94	43.3
	1	34	15.7
	2	31	14.3*
	3	58	26.7
Item 58	0	92	42.4
	1	31	14.3*
	2	40	18.4
	3	54	24.9
Item 59	0	64	29.5
	1	6	2.8*
	2	37	17.1
	3	110	50.7
Item 60	0	87	40.1
	1	8	3.7*
	2	63	29.0
	3	59	27.2
Item 61	0	85	39.2
	1	10	4.6*

	2	65	30.0
	3	57	26.3
Item 62	0	79	36.4
	1	14	6.5*
	2	55	25.3
	3	69	31.8
Item 63	0	83	38.2
	1	21	9.7*
	2	26	12.0*
	3	87	40.1
Item 64	0	73	33.6
	1	9	4.1*
	2	19	8.8*
	3	116	53.5

* floor effect

Table S5. Ceiling-Floor Effect Dimension E of the GMFM-88.

**Ceiling-Floor Effect Dimension E of the GMFM-88 (sample 217)**			
Dimension E	Response	Frequency	Percentage
Item 65	0	53	24.4
	1	5	2.3*
	2	4	1.8*
	3	155	71.4
Item 66	0	53	24.4
	1	5	2.3*
	2	4	1.8*
	3	155	71.4
Item 67	0	45	20.7
	1	3	1.4*
	2	4	1.8*
	3	165	76.0
Item 68	0	58	26.7
	1	4	1.8*
	2	3	1.4*
	3	152	70.0
Item 69	0	61	28.1
	1	7	3.2*
	2	5	2.3*
	3	144	66.4
Item 70	0	74	34.1
	1	2	.9*
	2	6	2.8*
	3	135	62.2
Item 71	0	111	51.2
	1	4	1.8*
	2	8	3.7*
	3	94	43.3
Item 72	0	82	37.8
	1	4	1.8*
	2	4	1.8*
	3	127	58.5
Item 73	0	103	47.5
	1	15	6.9*
	2	15	6.9*
	3	84	38.7
Item 74	0	127	58.5
	1	25	11.5*

	2	20	9.2*
	3	45	20.7
Item 75	0	112	51.6
	1	10	4.6*
	2	13	6.0*
	3	82	37.8
Item 76	0	113	52.1
	1	9	4.1*
	2	14	6.5*
	3	81	37.3
Item 77	0	141	65.0
	1	16	7.4*
	2	12	5.5*
	3	48	22.1
Item 78	0	85	39.2
	1	1	.5*
	2	3	1.4*
	3	128	59.0
Item 79	0	85	39.2
	1	1	.5*
	2	3	1.4*
	3	128	59.0
Item 80	0	127	58.5
	1	33	15.2
	2	38	17.5
	3	19	8.8
Item 81	0	136	62.7
	1	15	6.9*
	2	26	12.0*
	3	40	18.4
Item 82	0	153	70.5
	1	18	8.3*
	2	23	10.6*
	3	23	10.6*
Item 83	0	154	71.0
	1	18	8.3*
	2	22	10.1*
	3	23	10.6*
Item 84	0	104	47.9
	1	11	5.1*
	2	11	5.1*

	3	91	41.9
Item 85	0	108	49.8
	1	17	7.8*
	2	18	8.3*
	3	74	34.1
Item 86	0	140	64.5
	1	11	5.1*
	2	15	6.9*
	3	51	23.5
Item 87	0	140	64.5
	1	13	6.0*
	2	14	6.5*
	3	50	23.0
Item 88	0	158	72.8
	1	1	.5*
	2	9	4.1*
	3	49	22.6

*floor effect
